# Identification and Computational Analysis of *BRCA1* Variants in Mexican Women from Jalisco, Mexico, with Breast and Ovarian Cancer

**DOI:** 10.3390/medsci14040450

**Published:** 2026-08-01

**Authors:** Martha Patricia Gallegos-Arreola, Asbiel Felipe Garibaldi-Ríos, Ingrid Patricia Dávalos-Rodríguez, María Teresa Magaña-Torres, Luis E. Figuera, Guillermo Moisés Zúñiga-González, Belinda Claudia Gómez-Meda, Blanca Miriam Torres-Mendoza, Raquel Villegas-Pacheco, René Gómez-Cerda, Julio César Cárdenas-Valdez, Sergio Osvaldo Meza-Chavolla, Mónica Alejandra Rosales-Reynoso, Wenceslao Guillermo Ángeles-Bueno, María José Gómez-Villegas, Daniela del Rocío Panduro Espinoza, José Elías García-Ortiz

**Affiliations:** 1 División de Genética, Centro de Investigación Biomédica de Occidente (CIBO), Centro Médico Nacional de Occidente (CMNO), Instituto Mexicano del Seguro Social (IMSS), Guadalajara 44340, Jalisco, Mexico; marthapatriciagallegos08@gmail.com (M.P.G.-A.); garibaldi4757@alumnos.udg.mx (A.F.G.-R.); luisfiguera@yahoo.com (L.E.F.); 2 Doctorado en Genética Humana, Centro Universitario de Ciencias de la Salud (CUCS), Universidad de Guadalajara (UdeG), Guadalajara 44340, Jalisco, Mexico; 3 División de Medicina Molecular, Centro de Investigación Biomédica de Occidente (CIBO), Centro Médico Nacional de Occidente (CMNO), Instituto Mexicano del Seguro Social (IMSS), Guadalajara 44340, Jalisco, Mexico; 4 Instituto de Genética Humana “Dr. Enrique Corona Rivera”, Departamento de Biología Molecular y Genómica, Centro Universitario de Ciencias de la Salud (CUCS), Universidad de Guadalajara (UdeG), Guadalajara 44340, Jalisco, Mexico; 5 Laboratorio de Inmunodeficiencias Humanas y Retrovirus, División de Neurociencias, Centro de Investigación Biomédica de Occidente (CIBO), Centro Médico Nacional de Occidente (CMNO), Instituto Mexicano del Seguro Social (IMSS), Guadalajara 44340, Jalisco, Mexico; 6 Departamento de Disciplinas Filosófico Metodológicas, Centro Universitario de Ciencias de la Salud (CUCS), Universidad de Guadalajara (UdeG), Guadalajara 44340, Jalisco, Mexico; 7 Unidad Médica de Alta Especialidad, Hospital de Gineco-Obstetricia, Centro Médico Nacional de Occidente (CMNO), Instituto Mexicano del Seguro Social (IMSS), Guadalajara 44340, Jalisco, Mexico; 8 Unidad de Detección y Diagnóstico Clínica de Mama, Instituto Mexicano del Seguro Social (IMSS), Guadalajara 44340, Jalisco, Mexico

**Keywords:** *BRCA1* gene, pathogenic *BRCA1* variants, breast cancer, ovarian cancer, genetic variation, computational biology

## Abstract

Background. Breast and ovarian cancer are among the most common neoplasms in women, and germline variants in the *BRCA1* gene markedly increase the risk of developing them. Despite existing studies, the frequency and spectrum of *BRCA1* variants in women from Jalisco, Mexico, remain underexplored. Objective. To identify and characterize *BRCA1* variants in a cohort of Mexican women with breast and ovarian cancer, and to assess their functional and splicing consequences through computational analysis. Methodology. Genomic DNA from 228 women with breast and/or ovarian cancer, selected by clinical criteria suggestive of hereditary cancer, was analyzed by next-generation sequencing. The functional impact of point variants was predicted with Ensembl VEP, SIFT, PolyPhen-2, REVEL, CADD, and AlphaMissense, and their effect on splicing was evaluated with SpliceAI, using the MANE Select canonical transcript (NM_007294.4). Results. *BRCA1* variants were identified in 14.0% of this screening-enriched cohort, with carrier proportions of 16.2% in ovarian and 13.6% in breast cancer; these figures reflect a selected series and do not represent population prevalence. Breast cancer carriers showed a younger age at diagnosis, a higher proportion of the triple-negative phenotype, and a stronger family history. Fourteen-point variants and four large rearrangements were detected, with a predominance of truncating loss-of-function alterations. The recurrent missense variant c.5123C>A (p.Ala1708Glu), located in the BRCT1 domain, was the most relevant finding, present in 28.1% of carriers (9/32). The computational analysis did not reclassify variants on its own but provided complementary evidence (PP3/BP4) consistent with current classifications: it allowed reannotation of c.5243G>A from nonsense to missense, supported the pathogenic nature of the intronic splice variant c.4987-3C>A, and was concordant with the likely benign interpretation of c.2735A>G, while c.3367G>T remained of uncertain significance. Conclusions. This cohort from Western Mexico harbors a distinctive spectrum of *BRCA1* variants, including the recurrent c.5123C>A variant, which may reflect a regional founder effect warranting haplotype analysis. These findings underscore the need for local evidence and for genetic panels adapted to the Mexican population, to enable accurate and equitable variant interpretation and access to targeted therapies such as PARP inhibitors.

## 1. Introduction

Worldwide, malignancies of the breast and ovary represent two of the most prevalent and deadly cancer types affecting females [1]. The etiology of these neoplasms involves a complex interaction between environmental exposures and inherited susceptibility [2]. Individuals carrying pathogenic germline alterations in the BRCA1 gene face a dramatically elevated lifetime predisposition to breast and ovarian carcinomas compared with the general population [2,3]. Furthermore, defective BRCA1 function has been linked to increased susceptibility to additional malignancies, such as pancreatic and prostate tumors [4].

Situated on chromosome 17q21, the BRCA1 gene is essential for genome preservation, primarily governing the repair of DNA double-strand breaks through the homologous recombination pathway. Structurally, the encoded protein features key conserved regions, notably the N-terminal RING finger domain, which confers E3 ubiquitin ligase activity, and the C-terminal BRCT domains, required for phosphopeptide binding and the assembly of DNA repair complexes [5,6,7]. Deleterious mutations disrupt these cellular mechanisms, impairing homologous recombination fidelity and driving the genomic instability that underlies cancer development [8,9,10].

Genetic sequencing has enabled the identification of numerous germline variants in *BRCA1*, which are typically classified as benign, likely benign, of uncertain significance, likely pathogenic, or pathogenic across various databases [11]. However, many variants are classified inconsistently across databases, as their clinical relevance lacks consensus [12,13]. The integration of bioinformatics tools to evaluate the impact of variants on the protein and on splicing provides valuable, complementary evidence to refine their classification, especially for variants of uncertain significance (VUS) [14,15,16,17].

Although studies have examined the frequency and spectrum of *BRCA1* variants across different regions of Mexico [17,18], to our knowledge, none have specifically focused on the population of the state of Jalisco. This regional focus is relevant because Mexico exhibits marked genetic heterogeneity, with admixture patterns that vary across states [19,20]. Jalisco, in particular, exhibits a distinctive profile with a relatively balanced proportion of European and Native American ancestry [21]. Therefore, analyzing this population using a combination of genetic and in silico approaches provides insight into the country’s genetic diversity and may reveal clinically relevant variants not previously characterized in other Mexican cohorts.

In this study, we analyzed the *BRCA1* gene variants identified in Mexican women with breast and ovarian cancer. Our objective was to characterize the frequency and spectrum of these variants in this population and to perform computational analyses to evaluate their functional significance, impact on splicing, and potential consequences for the BRCA1 protein.

## 2. Materials and Methods

### 2.1. Patients

Our study evaluated female Mexican patients aged 18 years or older who had a histopathologically confirmed diagnosis of breast or ovarian malignancy and a clinical presentation suggestive of hereditary cancer predisposition. All participants were native to the state of Jalisco, Mexico. Eligibility required fulfilling at least one established clinical criterion: early disease onset (diagnosed prior to age 50), a strong familial history of associated neoplasms (including breast, ovarian, pancreatic, or prostate cancer in first- or second-degree relatives), or the presence of multiple primary tumors.

The study was conducted according to the guidelines of the Declaration of Helsinki and Local Ethics and Research Committees (1305, CIBO, IMSS) (protocol code R-2022-1305-114 on 22 December 2022). Informed consent was obtained from all subjects involved in the study. Written informed consent has been obtained from the patient(s) to publish this paper.

### 2.2. Identification of BRCA1 Gene Variants

#### 2.2.1. Genomic DNA Extraction

Total genomic DNA was isolated from peripheral whole blood using the AmoyDx Blood/Bone Marrow kit (AmoyDX, Singapore) following the instructions provided by the manufacturer. Nucleic acid concentration and purity were measured via spectrophotometry using the Qubit 4 fluorometer (ThermoFisher Scientific, Waltham, MA, USA) and verified through agarose gel electrophoresis.

#### 2.2.2. BRCA1 Sequencing

Target enrichment covering all coding exons and adjacent intronic boundaries of the BRCA1 gene was accomplished using the AmpliSeq for Illumina BRCA Panel (Illumina, Inc., San Diego, CA, USA). Sequencing libraries were prepared in accordance with standard manufacturer protocols. Next-generation sequencing was subsequently performed on an Illumina MiSeq instrument (Illumina, Inc., San Diego, CA, USA), mapping reads against the canonical BRCA1 reference sequence (NM_007294.4).

#### 2.2.3. Bioinformatics Analysis and Variant Annotation

Raw sequence data alignment, variant calling, and initial functional annotation were executed using the ANDAS-Amoy v1.1.1 proprietary bioinformatics pipeline. Identified variants were filtered using quality benchmarks and a minor allele frequency threshold of less than or equal to 0.01 based on population datasets, including gnomAD v4.1.1 and ClinVar. Annotated parameters comprised genomic coordinates, predicted coding consequences, and registration in public clinical databases.

#### 2.2.4. Variant Classification and Evaluation

Clinical variant curation was conducted adhering to the joint consensus criteria established by the American College of Medical Genetics and Genomics and the Association for Molecular Pathology (ACMG/AMP) [22]. Alterations were stratified into five tiers: benign, likely benign, variant of uncertain significance, likely pathogenic, or pathogenic. Alterations categorized as benign or likely benign were excluded from the primary clinical report.

### 2.3. Computational Analysis of Variants

For the complementary functional characterization of point variants, in silico annotation was performed using the Variant Effect Predictor (VEP, Ensembl, GRCh38 assembly) v116 [23], utilizing the *BRCA1* MANE Select reference transcript (NM_007294.4/ENST00000357654.9; protein NP_009225.1). All annotations are reported with respect to this canonical transcript. Pathogenicity predictors at the protein level (SIFT v6.2.1 [24], PolyPhen-2 v2.2.3 [25], REVEL v4.7c [26], CADD v.17 [27], and AlphaMissense [28]) and splicing impact predictors (SpliceAI v1.3.1 and Pangolin v1.0.1) were integrated; the latter were queried through SpliceAI Lookup in unmasked score mode. This specific panel of tools was selected to combine traditional algorithms for historical comparability (SIFT and PolyPhen-2), established ensemble algorithms endorsed by clinical variant curation frameworks (REVEL and CADD), and advanced deep learning models (AlphaMissense, SpliceAI, and Pangolin). While consensus meta-predictors such as BayesDel offer high clinical utility, we prioritized REVEL as our primary ensemble tool to prevent redundancy and score collinearity, given that our pipeline already evaluated several underlying individual algorithms independently.

The interpretation thresholds were: deleterious SIFT < 0.05; probably damaging PolyPhen-2 > 0.85; pathogenic REVEL > 0.5 (intermediate zone 0.5–0.7); likely pathogenic AlphaMissense > 0.564; deleterious CADD > 20 (Phred scale); and SpliceAI/Pangolin with strong impact ≥ 0.5 and possible impact between 0.2 and 0.5. These predictions were used as complementary computational evidence (ACMG/AMP criteria PP3/BP4). Frameshift and nonsense variants, whose truncating effect is established (PVS1 criterion), were not subjected to missense predictors.

The location of each variant relative to the functional domains of the BRCA1 protein was determined based on UniProt annotations [29] (identifier P38398): RING zinc finger domain (amino acids 24–65), PALB2 interaction region (1397–1424), and the two BRCT domains (BRCT1, 1642–1736; BRCT2, 1756–1855). The distribution of variants along the protein and the graphical comparison of predictor scores were represented using the ggplot2 package in R (v4.2.1).

### 2.4. Statistical Analysis

Non-parametric continuous data were reported as medians with interquartile ranges (IQR) and evaluated using the Mann–Whitney U test. Categorical variables were expressed as frequencies and relative proportions of available cases, with group differences assessed via Fisher’s exact test, generating odds ratios (OR) and corresponding 95% confidence intervals (CI). Missing values were managed through available-case analysis without data imputation. To account for multiple testing, *p*-values were adjusted using the Benjamini–Hochberg false discovery rate method (q-values). For the ovarian subgroup (n = 6 carriers), identical statistical procedures were applied; however, due to limited sample size, these evaluations are treated strictly as exploratory and hypothesis-generating. All computational analyses were executed in R software (version 4.2.1) adopting a two-sided significance threshold of 0.05.

## 3. Results

### 3.1. Cohort Description

A cohort of 228 patients with breast (n = 191) and ovarian (n = 37) cancer was analyzed. Thirty-two *BRCA1* variant carriers (26 with breast cancer and 6 with ovarian cancer) and 196 non-carriers (165 with breast cancer and 31 with ovarian cancer) were identified. The frequency of *BRCA1* carriers was 13.6% in the breast cancer cohort and 16.2% in the ovarian cancer cohort. These figures correspond to a series enriched by screening criteria and do not represent population prevalence.

### 3.2. Clinicopathological Characteristics According to Carrier Status

#### 3.2.1. Breast Cancer

*BRCA1* carriers presented a significantly lower age at diagnosis than non-carriers (median 39 years, IQR 33–43 vs. 47 years, IQR 38–58; Mann–Whitney U test, *p* = 0.001). Diagnosis before 40 years of age was more frequent in carriers (56.0% vs. 32.9%; OR 2.59, 95% CI 1.10–6.09; *p* = 0.042). The triple-negative subtype was markedly predominant in carriers (83.3% vs. 45.0%; OR 6.11, 95% CI 2.00–18.69; *p* = 0.001), as was a family history of cancer (70.0% vs. 36.2%; OR 4.11, 95% CI 1.48–11.36; *p* = 0.006). In contrast, advanced clinical stage (III–IV) was not associated with carrier status (OR 0.80, 95% CI 0.32–1.99; *p* = 0.819). The complete set of characteristics is presented in Panel A of Table 1. After the Benjamini–Hochberg correction, the associations with the triple-negative subtype (q = 0.003), age at diagnosis (q = 0.003), and family history of cancer (q = 0.011) remained significant. The association with diagnosis before age 40 remained borderline (q = 0.053), consistent with information loss from dichotomizing age. Consequently, age is reported as a continuous variable in the main analysis.

#### 3.2.2. Ovarian Cancer

In the ovarian cohort, all 6 *BRCA1* carriers presented exclusively with high-grade serous carcinoma (100%), compared with 83.9% in non-carriers, with a similar median age at diagnosis (52 vs. 54 years). Exploratory tests were performed (Mann–Whitney U test for age and Fisher’s exact test for categorical variables), which showed no statistically significant differences between carriers and non-carriers in any of the evaluated variables (all *p*-values ≥ 0.117), and no association was maintained after Benjamini–Hochberg correction (all q-values ≥ 0.59). The apparent difference in greatest magnitude was for lymph node involvement (50.0% in carriers vs. 17.2%; OR 4.80, 95% CI 0.74–31.1; *p* = 0.117), albeit with an extremely wide confidence interval. Given the small number of carriers (n = 6), these comparisons lack statistical power and are presented solely as exploratory and hypothesis-generating (Panel B of Table 1). The response to platinum-based treatment was heterogeneous among carriers (complete response, partial response, and no response in 2 cases each).

### 3.3. Frequency and Spectrum of BRCA1 Variants

Among the 32 carriers, 14 point variants and 4 large rearrangements (deletions of one or more exons) were identified. The spectrum included frameshift, nonsense, missense, and splice-site variants, distributed across the RING, central, and BRCT domains of the protein. The details are presented in Table 2.

The most relevant finding was the missense variant c.5123C>A (p.Ala1708Glu), located in the BRCT1 domain, identified in 9 of the 32 carriers (28.1%): 5 with breast cancer and 4 with ovarian cancer.

The c.68_69del (p.Glu23ValfsTer17) variant, corresponding to the 185delAG founder variant of the RING domain, was identified in 4 breast cancer carriers. Three other variants were observed in two carriers each (c.2433del, c.3759_3760del, and c.3648dup). The four large deletions (exons 2–24, 8–11, 9–12, and 16–17) were detected in the breast cancer cohort.

#### Distribution of Variants by Functional Domain

**Figure 1:**
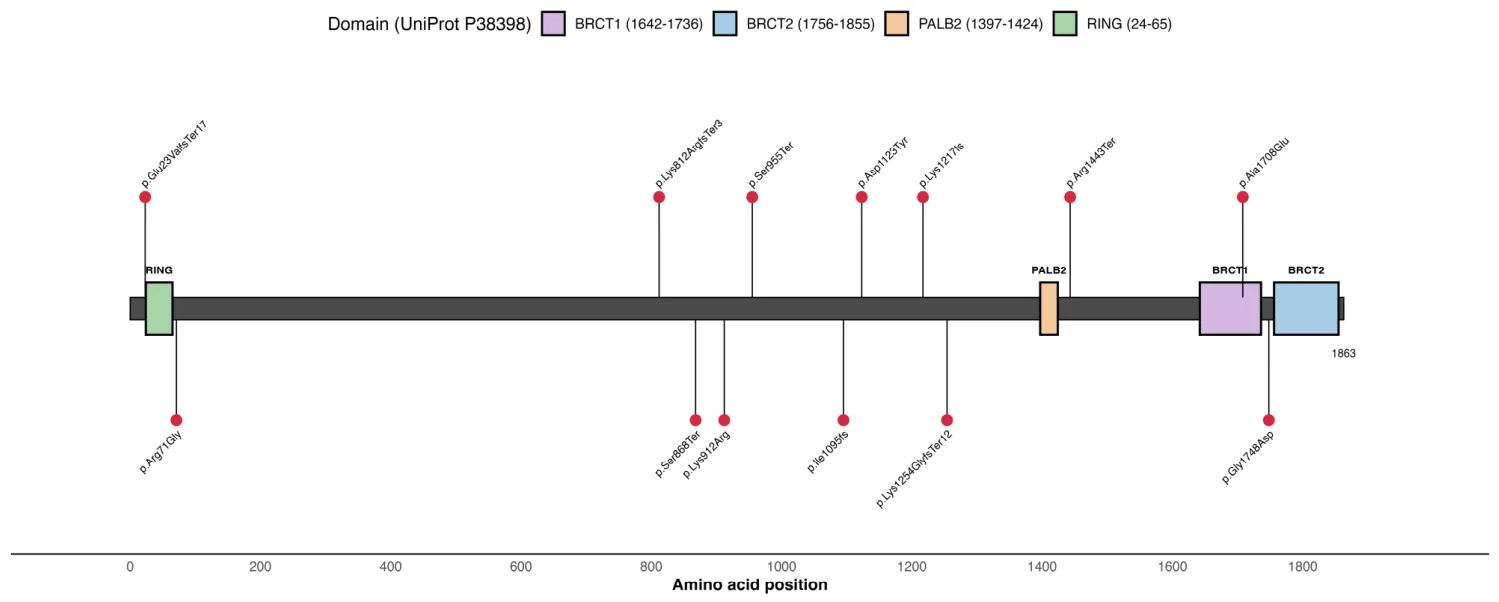
Distribution of the *BRCA1* point variants identified in the cohort, mapped onto the BRCA1 protein (UniProt P38398, 1863 aa). Domains: RING-type zinc finger (24–65), PALB2-interaction region (1397–1424), and the two BRCT domains (BRCT1 1642–1736; BRCT2 1756–1855. The intronic splice variant c.4987-3C>A and the four large rearrangements (CNVs), which lack a single defined protein position, are listed in Table 2.

The distribution of point variants along the BRCA1 protein (1863 amino acids; UniProt P38398) is depicted in Figure 1. Two variants were located at the amino-terminus corresponding to the RING domain (with E3 ubiquitin ligase activity): c.68_69del (p.Glu23ValfsTer17), immediately proximal to the domain, and c.211A>G (p.Arg71Gly), within it. Most of the variants (eight) were distributed across the extensive central region encoded by exon 10 (historically exon 11), a region without an annotated structured domain but containing multiple protein interaction sites. The c.4327C>T (p.Arg1443Ter) variant was located in the vicinity of the PALB2 interaction region (amino acids 1397–1424). Finally, the variants at the carboxyl-terminus, c.5123C>A (p.Ala1708Glu) in the BRCT1 domain and c.5243G>A (p.Gly1748Asp) in the linker region between BRCT1 and BRCT2, affect the phosphopeptide recognition region, which is functionally critical for the DNA damage response.

### 3.4. Computational Analysis

In silico annotation of the 14 point variants was performed using the MANE Select transcript, integrating protein pathogenicity and splicing impact predictors (Table 3). The five frameshift and three nonsense variants showed high CADD values (34–39) and a truncating consequence, consistent with their pathogenic classification (PVS1 criterion).

#### 3.4.1. Confirmation of the Recurrent Variant and Missense Variants

The recurrent variant c.5123C>A (p.Ala1708Glu) showed robust pathogenicity prediction (AlphaMissense 0.971; REVEL 0.831; CADD 27.7), with a SpliceAI value of 0.22 (possible skipping of exon 17). The c.5243G>A variant, initially reported as nonsense, was reannotated on the canonical transcript as missense p.Gly1748Asp (AlphaMissense 0.973; REVEL 0.732). The c.211A>G (p.Arg71Gly) variant exhibited a dual predicted mechanism: missense pathogenicity (AlphaMissense 0.901; REVEL 0.866; CADD 34) and a strong impact on splicing (SpliceAI 0.64; in-frame skipping of exon 4).

The intronic variant c.4987-3C>A, at the acceptor site of intron 16, obtained a prediction of strong impact on splicing (SpliceAI 0.73; Pangolin 0.80), with a concordant prediction of exon 16 skipping (88 bp) and a shift in the reading frame, which defines a loss-of-function mechanism.

#### 3.4.2. In Silico Pathogenicity and Splicing Predictions

The c.2735A>G (p.Lys912Arg) variant presented concordant predictions toward a benign effect across all predictors (AlphaMissense 0.082; REVEL 0.408; CADD 5.44; SIFT tolerated; PolyPhen-2 benign; PrimateAI-3D 0.03; SpliceAI 0.00), which provides computational evidence of a benign impact (BP4 criterion) and supports its reclassification to likely benign. In contrast, the c.3367G>T (p.Asp1123Tyr) variant showed conflicting evidence (AlphaMissense 0.129 and PrimateAI-3D 0.36 benign, versus SIFT 0 and PolyPhen-2 0.817 deleterious; REVEL intermediate 0.545); therefore, it remains a VUS (variant of uncertain significance).

**Figure 2:**
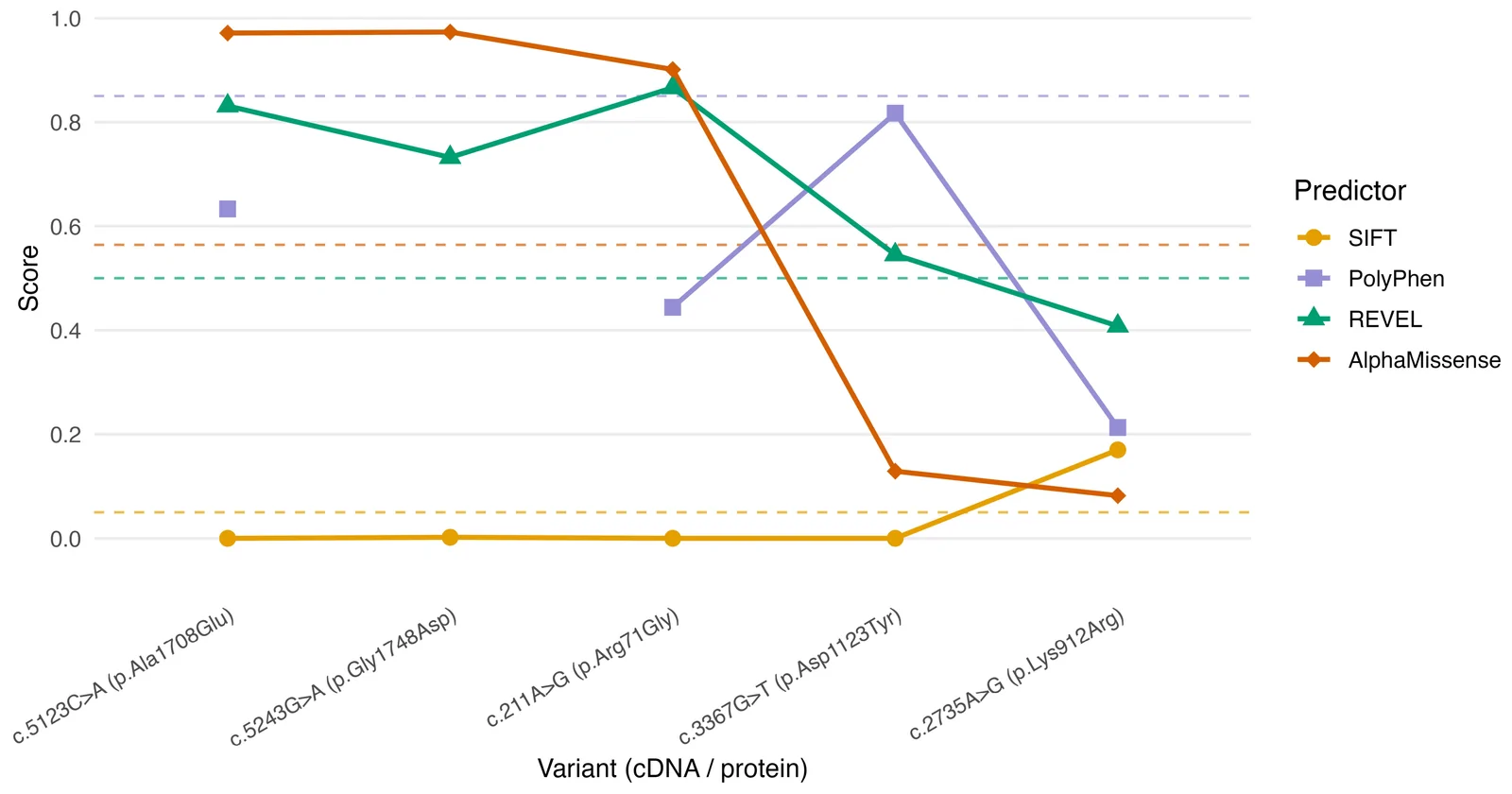
In silico pathogenicity scores of the five BRCA1 missense variants according to SIFT, PolyPhen-2, REVEL and AlphaMissense. Dashed lines, color-matched to their corresponding predictor, indicate the deleterious-prediction cutoff for each platform (SIFT 0.05, PolyPhen-2 0.85, REVEL 0.5, AlphaMissense 0.564). Note that SIFT scores are inversely oriented, with lower values indicating a more deleterious prediction.

The graphical comparison of the protein pathogenicity predictors for the five missense variants is shown in Figure 2, where the concordant or discordant behavior of each algorithm relative to its thresholds is evident. Variants c.5123C>A, c.5243G>A, and c.211A>G exceeded the pathogenicity thresholds across the majority of the predictors, whereas c.2735A>G remained below all of them, consistent with its reclassification to likely benign. The c.3367G>T variant exhibited the discordant pattern characteristic of variants of uncertain significance: above the threshold in PolyPhen-2 but below it in AlphaMissense, which precludes a conclusive reclassification.

## 4. Discussion

This investigation characterized the germline *BRCA1* mutational landscape in a cohort of female breast and ovarian cancer patients from Jalisco, Mexico, pairing clinical phenotypes with comprehensive computational variant evaluations. A total of 14 distinct point alterations and four exonic deletions were identified within this screening-enriched cohort. Consequently, the observed carrier proportions (13.6% in breast cancer and 16.2% in ovarian cancer) reflect the frequency within a clinically selected high-risk group rather than the general population prevalence. Our results should therefore be interpreted as an overview of the regional mutational spectrum rather than an epidemiological measure of *BRCA1* burden.

The clinicopathological characteristics of the breast cancer carriers were consistent with the phenotype classically associated with *BRCA1* [30]. Carriers presented a significantly younger age at diagnosis (median of 39 vs. 47 years), a marked predominance of the triple-negative subtype (83.3% vs. 45.0%; OR 6.11), and a higher frequency of a family history of cancer (70.0% vs. 36.2%; OR 4.11), associations that remained significant after correction for multiple comparisons. These results are consistent with the national literature, for instance Villarreal-Garza et al. [31] have consistently described that *BRCA*-associated breast cancer in Mexican women is characterized by an early age at diagnosis and a high proportion of triple-negative tumors, in the context of a population with a younger age of breast cancer presentation than that reported in populations of European descent [32]. The strong association with the triple-negative subtype observed in our series reinforces the relevance of offering *BRCA1* germline testing to Mexican patients with triple-negative breast cancer, regardless of age or family history, as recommended by current guidelines [33].

In the ovarian cancer cohort, all six carriers uniformly presented with high-grade serous carcinoma, the histological subtype characteristically linked to homologous recombination deficiency [34]. However, given the small number of carriers, comparative analyses in this subgroup were conducted using an exploratory, hypothesis-generating approach; no association reached statistical significance or withstood correction for multiple comparisons, and the confidence intervals were very wide. Therefore, the numerical differences observed, such as the higher frequency of lymph node involvement in carriers, must be interpreted with caution and require confirmation in larger series. To overcome this statistical limitation and reach sufficient statistical power in future studies, regional multicenter collaborations are currently being planned. Expanding recruitment across additional reference hospitals and oncology centers in Western Mexico will allow the assembly of a sufficiently powered ovarian cancer cohort to confirm these exploratory clinicopathological findings and establish robust genotype–phenotype correlations in our population.

In general, the importance of identifying *BRCA1* variant carriers with breast and ovarian cancer also lies in its direct therapeutic implications, given that these patients are candidates to receive PARP inhibitors [35], a treatment option whose equitable access remains a challenge in Latin American health systems [36,37].

In total, a spectrum of 14 distinct germline variants was identified in 32 carriers (26 with breast cancer and 6 with ovarian cancer). The mutational profile was characterized by a high burden of truncating loss-of-function alterations, with frameshift variants being the most frequent pathogenic mechanism, accounting for 11 patients. Notable within this group is the recurrent variant in the RING domain c.68_69del (p.Glu23ValfsTer17), identified in four breast cancer patients. Historically known as 185delAG, this alteration is a well-characterized founder mutation with a significant prevalence in Hispanic populations, reinforcing the influence of historical admixture on the genomic profile of our cohort [38].

The most relevant finding of the series was the recurrence of the c.5123C>A (p.Ala1708Glu) variant, present in nine carriers (five with breast cancer and four with ovarian cancer). This variant, located in the BRCT1 domain, which is critical for phosphopeptide recognition in the DNA damage response [7], showed a computational profile consistent with a deleterious effect (AlphaMissense 0.971; REVEL 0.831; CADD 27.7), with additional evidence of a potential impact on splicing (SpliceAI 0.22). Previous reports have identified it in breast cancer patients in Mexico [39], Spain [40] and Colombia [41]. This geographical distribution suggests a probable shared ancestral origin, possibly Iberian, with subsequent dispersion into Latin America. However, its recurrence in our specific cohort opens the possibility of a regional founder effect or a demographic expansion in the population of western Mexico. Since these hypotheses cannot be confirmed without a shared haplotype analysis, this proposition should be considered preliminary and a starting point for future population-based research. Clinically, patients carrying the c.5123C>A variant in our cohort exhibit the classic phenotype associated with pathogenic *BRCA1* alterations, characterized by early age at diagnosis, triple-negative breast tumors, and high-grade serous ovarian carcinomas. There is currently no clinical evidence indicating that this specific variant confers a worse prognosis or inferior survival outcome compared with canonical truncating mutations. Because the p.Ala1708Glu substitution severely impairs the BRCT1 domain and disrupts homologous recombination, tumors driven by this alteration behave functionally like standard loss-of-function mutations. Consequently, from a clinical management perspective, these patients retain the expected sensitivity to platinum-based chemotherapy and PARP inhibitors, which is the main determinant of clinical outcome.

When comparing our results with cohorts from other regions of Mexico, we observed important differences in the mutational spectrum. Although the overall frequency of *BRCA1* carriers in our series (14.0%) falls within the range reported in Mexican breast cancer cohorts (4.3% to 23% depending on patient selection and tumor phenotype) [39,42], the distribution of specific alterations varies considerably by geographic region. Research conducted primarily in central Mexico has consistently shown that the regional *BRCA1* spectrum is dominated by the ex9-12del founder mutation, which accounts for 30% to 41% of all pathogenic *BRCA1* variants in those cohorts [42,43]. In contrast, large rearrangements represented only 12.5% of the carriers (4/32) in our population from Jalisco, and the ex9-12del variant was identified in just a single patient.

In our series, the mutational profile is characterized instead by the high frequency of the c.5123C>A (p.Ala1708Glu) variant, present in 28.1% of the carriers (9/32). While this missense variant has been previously reported in studies from Central and Northern Mexico [17,39], its frequency in those populations is substantially lower. At the same time, our patients share well-known recurrent alterations reported across the country, such as the c.68_69del (p.Glu23ValfsTer17) variant, which was present in 12.5% of our carriers and reflects the historical European admixture found throughout Latin America [38]. This comparison confirms that the genetic substructure of the Mexican population [19,20,21] leads to distinct regional profiles of *BRCA1* variants. Consequently, diagnostic strategies and genetic panels need to consider not only national data, but also the specific distribution of variants across different regions of the country.

In addition to point variants and small deletions, it is important to highlight the detection of large rearrangements (exonic deletions) in four patients from the breast cancer cohort. The proportion of these structural variants emphasizes that genetic screening strategies in our population must obligatorily incorporate sensitive methodologies for CNVs, since relying exclusively on NGS without adequate bioinformatics analysis for CNVs, or without MLPA confirmation, would result in a considerable false-negative rate [44].

Computational analysis facilitated the interpretation of several variants. Annotation using the MANE Select canonical transcript and the integration of splicing predictors was highly useful for interpreting c.4987-3C>A, an intronic acceptor site variant whose strong predicted impact on splicing (SpliceAI 0.73; Pangolin 0.80) and consequent loss of exon 16 support its pathogenic nature, illustrating the utility of incorporating splicing predictors alongside protein pathogenicity predictors. It is important to mention that, to our knowledge, this variant has not been reported in Mexico or Latin American countries. In contrast, the allele change C>G (c.4987-3C>G) has been reported in families from the Netherlands, where its pathogenicity was also concluded through functional studies [45,46].

In addition to the recurrent c.5123C>A variant, another dual-effect variant was identified in the RING domain: c.211A>G (p.Arg71Gly), classified as pathogenic in ClinVar. Its clinical interest lies in its combination of a missense effect and a splicing donor-site alteration, the latter confirmed by functional RNA studies that demonstrate an aberrant transcript with a premature stop codon. It is worth mentioning that the variant has been described in families with a history of ovarian cancer in Spain [47,48] and Brazil [49]. However, in our cohort, it was detected in a breast cancer patient, a finding consistent with a previous report in the Mexican population [18]. This phenotypic transition broadens the clinical spectrum associated with the variant and, coupled with its geographic distribution, suggests a probable shared Ibero-American ancestral origin. This underscores the importance of not restricting the analysis of these alterations to specific phenotypes.

The c.2735A>G (p.Lys912Arg) variant is currently listed as likely benign (although, at the time of its report, it was considered a VUS), consistent with the in silico evidence from our analysis, which showed uniformly benign predictions (BP4 criterion). At the opposite end, c.5243G>A (p.Gly1748Asp), located in the linker between the BRCT domains, has been consolidated as likely pathogenic, supported by functional genomic saturation editing assays (loss of function) and multifactorial likelihood analysis, findings that are consistent with our pathogenicity predictions (AlphaMissense 0.973; REVEL 0.732). Only c.3367G>T (p.Asp1123Tyr) remains a variant of uncertain significance, maintaining a pattern of discordant predictions (deleterious by PolyPhen-2 and SIFT vs. benign by AlphaMissense and PrimateAI-3D) that prevents a conclusive interpretation. It is important to mention that, to our knowledge, the c.2735A>G and c.5243G>A variants have not been previously reported in national or international studies. However, the data presented here provide evidence for their reclassification and consideration as benign variants, which is clinically relevant, given that in the patient’s initial report they were classified as VUS.

It should be emphasized that the aforementioned computational predictions constitute complementary evidence (PP3/BP4 criteria of the ACMG/AMP framework) and do not equate to a formal clinical reclassification, which requires the integration of additional lines of evidence under a multifactorial approach. In this context, the integration of data from Massively Parallel Assays (MAVEs) represents a critical strategy for resolving unclassified variants and variants of uncertain significance, such as c.3367G>T. Unlike computational algorithms, validated MAVE platforms, including saturation genome editing and high-throughput functional assays measuring homology-directed repair, provide direct experimental evidence of protein function. Under current ACMG/AMP and ClinGen curation guidelines, robust MAVE datasets can fulfill the criteria for strong functional evidence (PS3/BS3). This experimental layer is especially valuable for resolving variants with conflicting computational predictions or those lacking sufficient familial segregation data. Furthermore, the interpretation of variants in Latin American populations faces a structural limitation stemming from the underrepresentation of these populations in reference genomic databases, which increases the proportion of VUS and delays their clinical resolution [50]. This phenomenon reinforces the importance of combining regional cohort reporting with high-throughput functional data such as MAVEs to improve diagnostic precision in underrepresented populations.

This study presents several limitations that should be considered. First, it is a single-center series enriched by screening criteria, so the frequencies cannot be extrapolated to the general population. Second, the size of the ovarian cancer cohort is small, which limits the power of comparative analyses in that subgroup. Third, the potential founder nature of the recurrent c.5123C>A variant could not be confirmed due to a lack of haplotype analysis. Fourth, the proposed reclassifications are based on computational evidence and require confirmation through functional and segregation studies. Finally, the conversion of exonic coordinates of large rearrangements to amino acid positions is approximate; therefore, they were not represented on the domain map. Despite these limitations, the study provides a detailed characterization of the *BRCA1* variant spectrum in a specific Mexican population, with implications for genetic counseling and variant interpretation in the region.

## 5. Conclusions

In this series of women from Jalisco with breast and ovarian cancer selected by hereditary cancer criteria, 14 point variants and four large rearrangements in *BRCA1* were identified. The c.5123C>A (p.Ala1708Glu) variant in the BRCT1 domain, present in 9 of the 32 carriers, was the central finding; its distribution in Mexico, Spain, and Colombia points to a shared Ibero-American origin and suggests a potential regional founder effect that will need to be confirmed through haplotype analysis.

Breast cancer carriers reproduced the classic *BRCA1* phenotype, featuring early diagnosis, a triple-negative predominance, and a family history of cancer, while ovarian cancer carriers uniformly presented with high-grade serous carcinoma. Computational analysis on the canonical transcript did not reclassify any variant on its own, but rather offered complementary evidence consistent with current classifications; it supported the pathogenic nature of the intronic splicing variant c.4987-3C>A, aligned with the pathogenicity of c.5123C>A and the likely benign interpretation of c.2735A>G, whereas c.3367G>T remained a variant of uncertain significance.

These results demonstrate that the population of western Mexico possesses a distinct spectrum of *BRCA1* variants that cannot be adequately interpreted using databases built on populations of European descent. Characterizing and reporting these variants is an indispensable condition for accurate genetic counseling, the design of diagnostic panels tailored to the Mexican population, and ensuring equitable access to targeted therapies such as PARP inhibitors.

## Figures and Tables

**Table 1:** Clinicopathological characteristics of breast and ovarian cancer patients according to *BRCA1* pathogenic variant carrier status.

Characteristic	*BRCA1*(+)	*BRCA1*(−)	*p*-Value	*q*-Value ‡	Statistic/OR
PANEL A. Breast cancer (*BRCA1*+ n = 26; non-carriers n = 165)					
Age at diagnosis, years—median (IQR)	39 (33–43)	47 (38–58)	0.001	0.003	Mann–Whitney U
Diagnosis < 40 years—n (%)	14 (56.0)	54 (32.9)	0.042	0.053	OR 2.59 (95% CI 1.10–6.09)
Molecular subtype	n (%)	n (%)			
Triple-negative	20 (83.3)	72 (45.0)			
Luminal A	0 (0.0)	29 (18.1)			
Luminal B	4 (16.7)	35 (21.9)			
HER2-positive	0 (0.0)	7 (4.4)			
Luminal (NOS)	0 (0.0)	14 (8.8)			
Other	0 (0.0)	3 (1.9)			
Triple-negative vs. others			0.001	0.003	OR 6.11 (95% CI 2.00–18.69)
Clinical stage	n (%)	n (%)			
0/in situ	0 (0.0)	1 (0.7)			
I	0 (0.0)	18 (11.8)			
II	15 (65.2)	72 (47.4)			
III	8 (34.8)	55 (36.2)			
IV	0 (0.0)	6 (3.9)			
Advanced (III–IV) vs. early (0–II)			0.819	0.819	OR 0.80 (95% CI 0.32–1.99)
Histology					
Invasive ductal carcinoma	20 (83.3)	149 (92.5)			
Lobular	1 (4.2)	3 (1.9)			
Other	3 (12.5)	9 (5.6)			
Family history of cancer	14 (70.0)	50 (36.2)	0.006	0.011	OR 4.11 (95% CI 1.48–11.36)
PANEL B. Ovarian cancer †	*BRCA1*(+) n = 6	*BRCA1* (-) n = 31			
Age at diagnosis, years—median (IQR)	52 (41–61)	54 (48–61)	0.697	0.697	Mann–Whitney U
	n (%)	n (%)			
High-grade serous histology	6 (100.0)	26 (83.9)	0.567	0.697	OR NE
Advanced stage (III–IV)	2 (33.3)	19 (61.3)	0.371	0.697	OR 0.32 (95% CI 0.05–2.00)
Family history of cancer	2 (33.3)	20 (64.5)	0.198	0.594	OR 0.28 (95% CI 0.04–1.75)
Positive lymph nodes	3 (50.0)	5 (17.2)	0.117	0.594	OR 4.80 (95% CI 0.74–31.1)
Complete platinum response	2 (33.3)	16 (53.3)	0.658	0.697	OR 0.44 (95% CI 0.07–2.76)

IQR, interquartile range; OR, odds ratio; CI, confidence interval; NOS, not otherwise specified; NE, not estimable (zero cell). Percentages were calculated over cases with available data—continuous variables: Mann–Whitney U test; categorical variables: Fisher’s exact test. ‡ *q*-value denotes the *p*-value after Benjamini–Hochberg correction for multiple comparisons, computed separately within each panel. † In the ovarian cohort (n = 6 carriers), comparisons were exploratory and hypothesis-generating only; no association reached statistical significance (all *p* ≥ 0.117) or survived Benjamini–Hochberg correction (all q ≥ 0.59), and confidence intervals are very wide owing to the small sample size. Exploratory analysis in cohorts enriched by screening criteria; associations may be influenced by selection bias.

**Table 2:** Spectrum of *BRCA1* variants identified in the cohort (n = 32 carriers).

cDNA (NM_007294.4)	Protein	Exon	Domain	Effect	ClinVar ^+^	Breast n	Ovary n	Total n
*c.5123C>A*	*p.Ala1708Glu*	18	BRCT1	Missense	Pathogenic	5	4	9
*c.68_69del*	*p.Glu23ValfsTer17*	2	RING	Frameshift	Pathogenic	4	0	4
*c.2433del*	*p.Lys812ArgfsTer3*	10	Central	Frameshift	Pathogenic	2	0	2
*c.3648dup*	*p.(Lys1217fs)*	10	Central	Frameshift	Pathogenic	1	1	2
*c.3759_3760del*	*p.Lys1254GlyfsTer12*	10	Central	Frameshift	Pathogenic	2	0	2
*c.211A>G*	*p.Arg71Gly*	5	RING	Missense/splicing	Pathogenic	1	0	1
*c.2603C>G*	*p.Ser868Ter*	10	Central	Nonsense	Pathogenic	1	0	1
*c.2735A>G*	*p.Lys912Arg*	10	Central	Missense	Likely Benign	0	1	1
*c.2864C>A*	*p.Ser955Ter*	10	Central	Nonsense	Pathogenic	1	0	1
*c.3285del*	*p.(Ile1095fs)*	10	Central	Frameshift	Pathogenic	1	0	1
*c.3367G>T*	*p.Asp1123Tyr*	10	Central	Missense	Conflicting classifications of pathogenicity	1	0	1
*c.4327C>T*	*p.Arg1443Ter*	12	Central	Nonsense	Pathogenic	1	0	1
*c.4987-3C>A*	*Intronic*	intron 16	BRCT	Splice-site	Conflicting classifications of pathogenicity	1	0	1
*c.5243G>A*	*p.Gly1748Asp*	18	BRCT1–BRCT2 linker	Missense	Pathogenic/Likely pathogenic	1	0	1
*Large rearrangements (CNV)*	exon deletions	2–24 *	Multiple	Large del.	Pathogenic	4	0	4

Reference transcript NM_007294.4 (protein NP_009225.1). Exon numbering follows the current MANE Select annotation (the historical exon 11 corresponds to exon 10). CNV, copy-number variant. The recurrent missense variant c.5123C>A (p.Ala1708Glu) in the BRCT1 domain was present in 9/32 carriers (28.1%). * Large rearrangements comprised four distinct multi-exon deletions (exons 2–24, 8–11, 9–12, and 16–17). ^+^ Retrieved from ClinVar on 10 June 2026.

**Table 3:** In silico pathogenicity and splicing predictions for the point variants identified in *BRCA1* (MANE Select transcript NM_007294.4).

cDNA	Protein	SIFT	PolyPhen	REVEL	AlphaMissense	CADD	SpliceAI	Interpretation
*c.5123C>A*	*p.Ala1708Glu*	0	0.633	0.831	0.971	27.7	0.22	Pathogenic (missense; possible splicing)
*c.5243G>A*	*p.Gly1748Asp*	0.002	—	0.732	0.973	24	0.01	Pathogenic (missense)
*c.211A>G*	*p.Arg71Gly*	0	0.444	0.866	0.901	34	0.64	Pathogenic (missense + splicing)
*c.3367G>T*	*p.Asp1123Tyr*	0	0.817	0.545	0.129	19.6	0.07	VUS (conflicting)
*c.2735A>G*	*p.Lys912Arg*	0.17	0.213	0.408	0.082	5.44	0.00	Likely benign (BP4)
*c.4987-3C>A*	Intronic	—	—	—	—	—	0.73	Pathogenic (splicing; exon 16 skip)
*c.4327C>T*	*p.Arg1443Ter*	—	—	—	—	35	0.01	Pathogenic (nonsense)
*c.2864C>A*	*p.Ser955Ter*	—	—	—	—	34	0.00	Pathogenic (nonsense)
*c.2603C>G*	*p.Ser868Ter*	—	—	—	—	38	0.00	Pathogenic (nonsense)

SIFT (deleterious < 0.05); PolyPhen-2 (probably damaging > 0.85); REVEL (pathogenic > 0.5; 0.5–0.7 intermediate); AlphaMissense (likely pathogenic > 0.564); CADD (Phred-scaled, deleterious > 20); SpliceAI (Δ score; ≥0.5 strong, 0.2–0.5 possible). Frameshift variants are not shown, as missense/splicing predictors do not apply; all are pathogenic (PVS1). c.2735A>G shows concordant benign predictions supporting reclassification toward likely benign (BP4); c.3367G>T remains a VUS owing to conflicting evidence.

## Data Availability

The original contributions presented in this study are included in the article. Further inquiries can be directed to the corresponding author.
